# Development of an Al-Based Web Diagnostic System for Phenotyping Psychiatric Disorders

**DOI:** 10.3389/fpsyt.2020.542394

**Published:** 2020-11-05

**Authors:** Yu-Wei Chang, Shih-Jen Tsai, Yung-Fu Wu, Albert C. Yang

**Affiliations:** ^1^Institute of Brain Science and Digital Medicine Center, National Yang-Ming University, Taipei, Taiwan; ^2^Department of Psychiatry, Taipei Veterans General Hospital, Taipei, Taiwan; ^3^Division of Psychiatry, School of Medicine, National Yang-Ming University, Taipei, Taiwan; ^4^Department of Psychiatry, Beitou Branch, Tri-service General Hospital, National Defense Medical Center, Taipei, Taiwan; ^5^Brain Medicine Center, Tao-Yuan Psychiatric Center, Tao-Yuan, Taiwan

**Keywords:** neuroimaging, classification, explainable deep neural network, schizophrenia, structural MRI

## Abstract

**Background:** Artificial intelligence (AI)-based medical diagnostic applications are on the rise. Our recent study has suggested an explainable deep neural network (EDNN) framework for identifying key structural deficits related to the pathology of schizophrenia. Here, we presented an AI-based web diagnostic system for schizophrenia under the EDNN framework with three-dimensional (3D) visualization of subjects' neuroimaging dataset.

**Methods:** This AI-based web diagnostic system consisted of a web server and a neuroimaging diagnostic database. The web server deployed the EDNN algorithm under the Node.js environment. Feature selection and network model building were performed on the dataset obtained from two hundred schizophrenic patients and healthy controls in the Taiwan Aging and Mental Illness (TAMI) cohort. We included an independent cohort with 88 schizophrenic patients and 44 healthy controls recruited at Tri-Service General Hospital Beitou Branch for validation purposes.

**Results:** Our AI-based web diagnostic system achieved 84.00% accuracy (89.47% sensitivity, 80.62% specificity) for gray matter (GM) and 90.22% accuracy (89.21% sensitivity, 91.23% specificity) for white matter (WM) on the TAMI cohort. For the Beitou cohort as an unseen test set, the model achieved 77.27 and 70.45% accuracy for GM and WM. Furthermore, it achieved 85.50 and 88.20% accuracy after model retraining to mitigate the effects of drift on the predictive capability. Moreover, our system visualized the identified voxels in brain atrophy in a 3D manner with patients' structural image, optimizing the evaluation process of the diagnostic results.

**Discussion:** Together, our approach under the EDNN framework demonstrated the potential future direction of making a schizophrenia diagnosis based on structural brain imaging data. Our deep learning model is explainable, arguing for the accuracy of the key information related to the pathology of schizophrenia when using the AI-based web assessment platform. The rationale of this approach is in accordance with the Research Domain Criteria suggested by the National Institute of Mental Health.

## Introduction

Advancement in computational strategy and the collection of big medical data have been contributing to an increasing interest in applying artificial intelligence (AI) to the medical field nowadays. Recent surge in the automated assessment of various medical diseases has suggested promising direction of improving the conventional workflow of the clinical practice. For example, AI-based medical applications have been consistently shown to successfully identify pathological patterns comparable or even superior to trained physicians (1, 2). The powerful pattern recognition adopted in these state-of-art AI algorithms can be crucial in delineating the complex pathophysiology of various medical diseases. Specifically, the AI algorithm may help identify subtle changes in brain images acquired from patients with mental disorders, which would tremendously support clinicians in the daily practice (3).

For diagnosis of major psychiatric disorder, the traditional approach is still exclusively based on phenomenology (4), leading to increasing interests in searching for objective biomarkers to different types of mental illnesses (5). Magnetic resonance imaging (MRI), which provides quantitative information of brain tissues, is one of the most used techniques for studying major psychiatric disorders. Conventional applications of machine learning techniques [e.g., support vector machines (SVM), random forest (RF)] are useful to perform prediction and discrimination tasks and have been commonly used for neuroanatomical investigations (6–10). However, these algorithms are unable to locate widespread patterns of abnormalities across the brain. Furthermore, limitations such as region-of-interest approaches or univariate analyses in conventional machine-learning models constantly lead to concerns about interpretability and generalizability (11). To tackle these difficulties, Chin et al. (12) introduced an anatomical regularized SVM classifier which yielded accuracy of 89.4% and demonstrated the utility of spatial and anatomical priors for structural MRI analyses. Apart from the conventional machine learning techniques, the neural network-based approaches have shown promising classification results when it is used with structural MRI, functional MRI, and diffusion tensor imaging (DTI) together (13–16). However, to our current knowledge, the deep-learning-based model with mere structural MRI data has not been fully developed so far, leaving it unclear about the classification capability for identifying psychiatric disorders and brain structural abnormalities as well as visualization of key deficits (17–19). Therefore, we applied the deep learning approach to identify diagnosis-specific brain region that can help differentiate schizophrenia and healthy controls. Given that brain imaging pathology in mental disorders is inconspicuous, developing explainable machine-learning models is inherently crucial, especially for indexing brain biomarkers in clinical practice with neuroimaging data (20).

In this study, we built an online website service for assessment of brain images based on an explainable deep learning algorithm (21). This AI-based web assessment system was developed for the purpose of classifying schizophrenia patients and healthy controls with structural MRI brain images. Furthermore, we aim to provide better interpretation from physician's point of view by visualizing the brain imaging pathology through web browser along with the analysis of structural deficits based on deep-learning findings. We anticipated this system to facilitate the exploitation of the promising AI technique for psychiatric applications.

## Methods and Materials

### Participants

**Figure 1:**
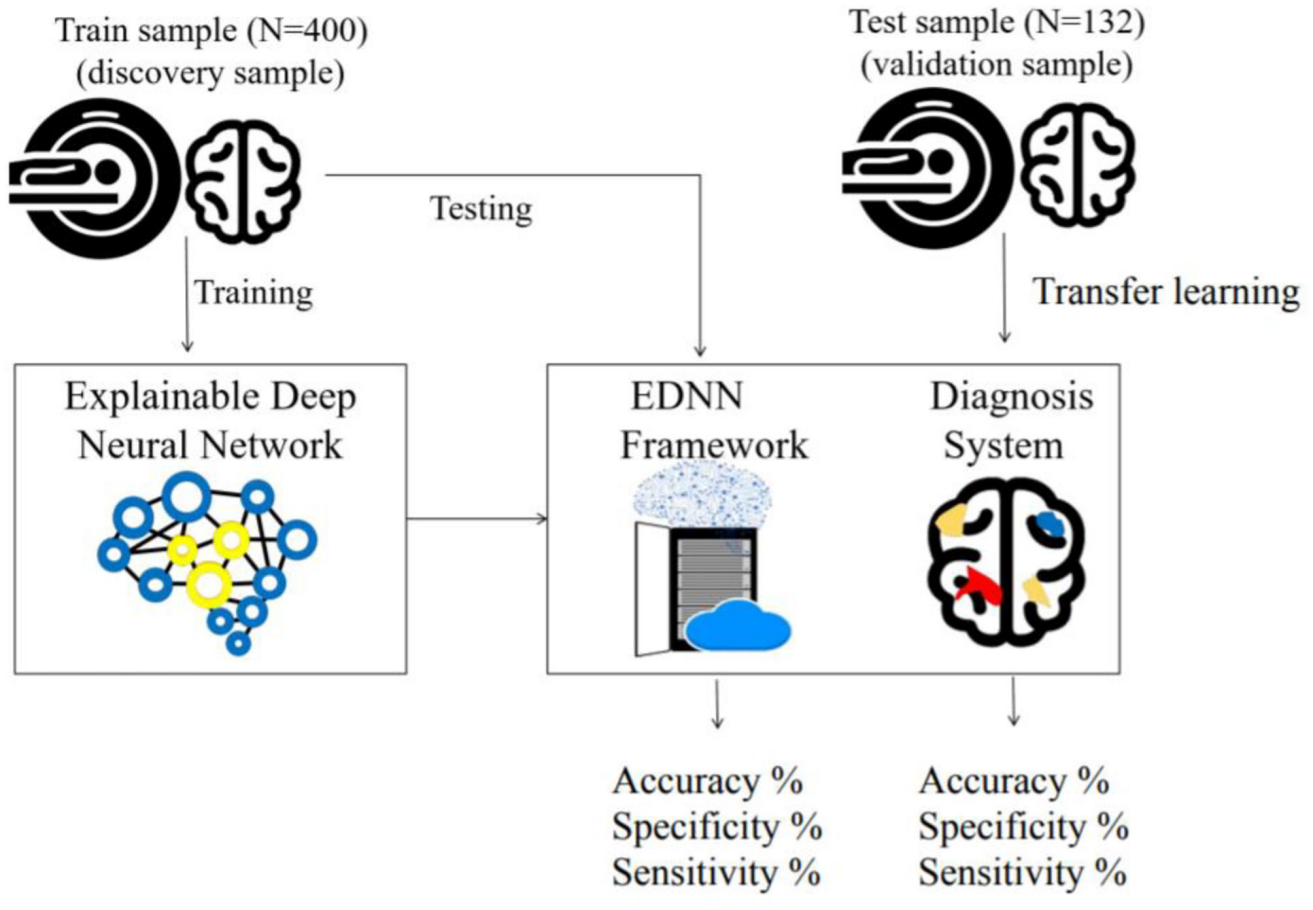
Framework of the train and test procedure: In the top left, the cohort is used to train the EDNN for the classification model. In the lower right box, a cartoon figure shows a trained model being deployed on a web server. The diagnostic result of the imaging data is visualized on the web browser. In the top right, the independent cohort is used to validate the model by transfer learning.

In this study, two independent cohorts with a total of 532 subjects from different institutions were included for model building and model transfer validation. First, the dataset for model building consisted of 200 patients with schizophrenia and 200 age- and sex-matched healthy controls from the discovery cohort “Taiwan Aging and Mental Illness” (TAMI). Data from 400 participants were divided into a training set consisting of 350 participants (87.50%) and a test set with 50 participants (12.50%). Second, the dataset for model evaluation and transfer learning comprised 88 patients and 44 health controls at Tri-Service General Hospital Beitou Branch, Taiwan. For clinical data evaluation, we applied the abovementioned model on the unseen data from 132 participants. The transfer learning was conducted by using the unseen dataset for model retraining. In line with the approach, data from 132 participants were divided into a training set consisting of 92 participants (69.69%) and a test set with 40 participants (30.31%). The training and test sets were randomly selected by matching participants based on diagnosis for the partitioning of data (Figure 1).

All schizophrenic patients were evaluated by the same protocol described previously (22). Healthy controls were recruited from the local communities through advertisements, and none of the them reported a history of neurological or psychiatric disorders. The study was approved by the Institutional Review Board of Taipei Veterans General Hospital (TVGH). Informed consent was obtained from all the participants before the MRI scanning. Tables 1, 2 shows demographic features of the participants.

**Table 1:** Demographics and clinical characteristics of participants.

	**TAMI**	**Beitou**
*N*	400	132
SCZ/HC	200/200	88/44
Male/Female	197/203	55/77
Age (Mean ± S.D)	43.6 ± 13.0	45.8 ± 13.1
Illness Duration, in Years (Mean ± S.D)	15.5 ± 10.9	22.8 ± 11.3
PANSS Total Score (Mean ± S.D)	40.8 ± 11.6	72.3 ± 15.6

**Table 2:** Further detailed information of schizophrenic participants from TAMI cohort (*N* = 200).

**Further detailed information**	**SCZ from TAMI (*N* = 200)**
Chlorpromazine Equivalent Dose (mg/day) (Mean ± S.D)	385.7 ± 329.6
Smoking Habits (Yes/No)	71/129
Onset Age (Mean ± S.D)	28.2 ± 10.1

### MRI Data Acquisition and Imaging Processing

Structural T1-weighted images were acquired at National Yang-Ming University with a 3.0-T Siemens Magnetom Tim Trio Scanner (Siemens AG, Erlangen, Germany) using a 3D magnetization-prepared rapid gradient echo (MPRAGE) sequence (TR = 2,530 ms, TE = 3.5 ms, TI = 1,100 ms, FOV = 256 mm, flip angle = 7) and a 12-channel head coil. The scanning protocol was the same as our prior reports (22–24).

Data were analyzed with DPARSF (V4.3) and SPM12 (Wellcome Trust Center for Neuroimaging, London, UK). T1 images were resliced (2 mm isotropic) and segmented into gray matter (GM), white matter (WM), and CSF tissue. Data preprocessing included slice timing, segmentation, and normalization into the Montreal Neurological Institute (MNI) stereotactic space.

The normalized GM and WM images served as the inputs per case for the web diagnostic system under the EDNN framework. In this approach, each subject's input images are considered as a data point in a high-dimensional space of anatomical information defined by GM or WM volumes.

### Neural Network and Web Diagnostic System

An explainable deep neural network (EDNN) with KL-L1 regularization is a classifier capable of identifying key structural deficits in schizophrenia (21). In this study, we adapted the EDNN framework to classify schizophrenia patients and healthy controls based on whole-brain gray matter and white matter densities (voxel-based morphometry, VBM).

The EDNN model is composed of a feature selection process and two fully connected layers with the KL-L1 regularization method.

$$\begin{array}{l} L_{KL-L1}\left(\theta \right)=\underset{i}{\sum }\|\bar{W_{i\cdot }}{\|}_{1}\log\frac{\|\bar{W_{i\cdot }}{\|}_{1}}{\varepsilon }+\left(1-\|\bar{W_{i\cdot }}{\|}_{1}\right)\\ \;\log\frac{1-\|\bar{W_{i\cdot }}{\|}_{1}}{1-\varepsilon }\\ \;I\left(X,Y\right)\;=\;\underset{x\epsilon X}{\sum }\underset{y\epsilon Y}{\sum }p\left(x,y\right)\;log\;p\left(y\;|\;x\right)\;-\;\underset{y\epsilon Y}{\sum }p\left(y\right)\;log\;p\left(y\right) \end{array}$$

mutual information analysis, *I*(*X, Y*), served as a feature selection process in the EDNN. This analysis was employed to reduce the dimensionality of neuroimaging data and to identify the key voxels that contribute to classification of schizophrenia. After implementing this selection of features, corresponding identified brain voxels that were associated with diagnosis label are then used to perform deep neural network learning and to investigate the performance for later classification.

**Figure 2:**
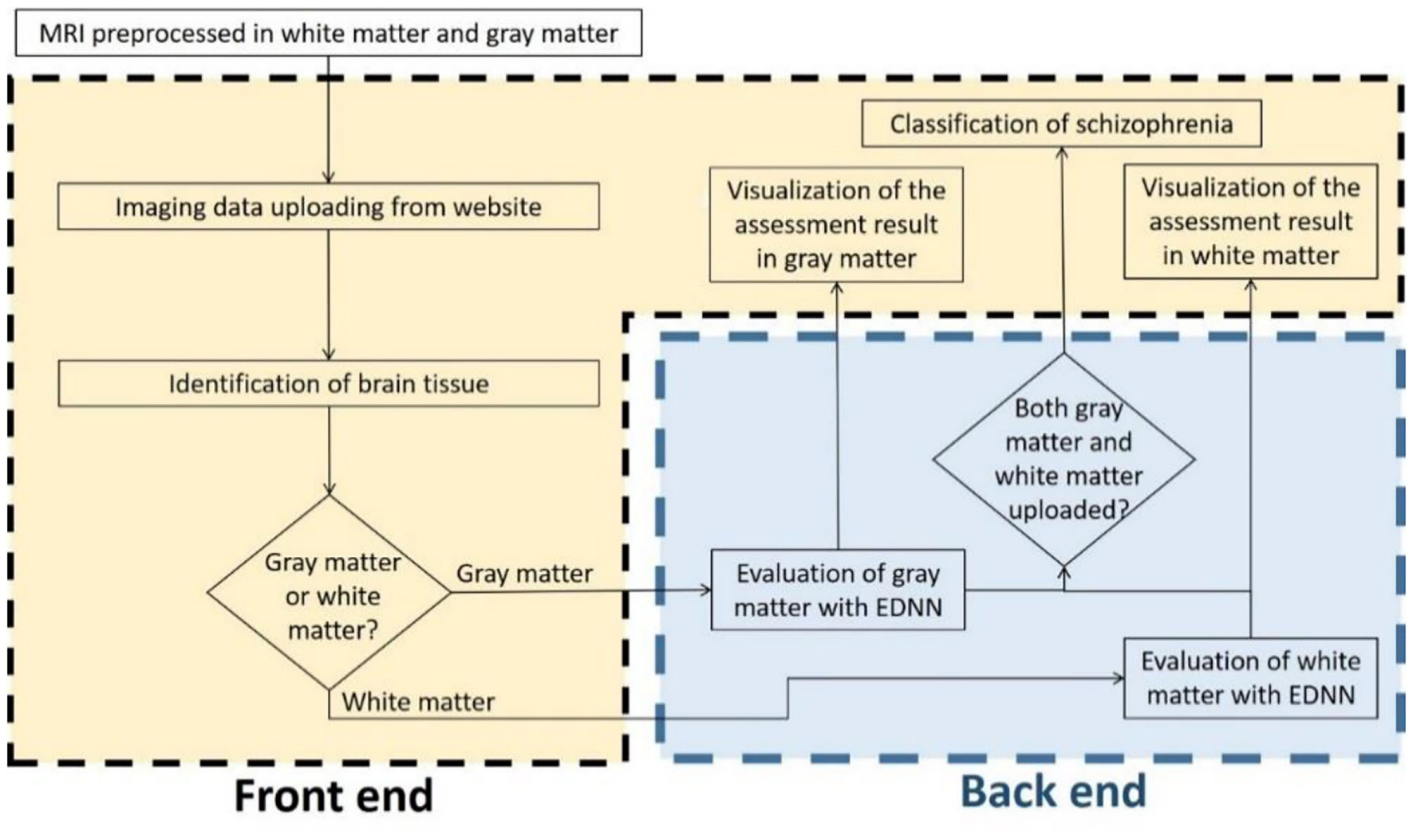
Flowchart of the assessment system. The evaluation of brain imaging data is divided separately into two-fold regarding the types of brain tissue. The diagnostic system individually evaluates the uploading imaging data in case of the uploading imaging data being identified as a gray matter or white matter imaging data, and then the system provides the analysis in brain morphometry to the front-end accordingly. As soon as both of the white matter and gray matter are uploaded and evaluated, a classification diagnosis of healthy and schizophrenia is intuitively obtained and presented to the end user.

To develop a systemic diagnostic platform for differentiating the information of brain imaging data in healthy and schizophrenia patients, we have proposed an AI-based web diagnostic system (Figure 2). The system consists of the web server and the imaging diagnostic database.

The server is constructed with Node.js and Tensorflow.js. Node.js is a platform on Chrome's JavaScript runtime for building scalable network applications. Node.js has been tested to yield better efficiency than PHP and Python-Web (25). Tensorflow.js provides flexible API in JavaScript, and it is part of the TensorFlow ecosystem. On the server side, we integrated feature selection and deep-learning framework (21) for the EDNN model. The model is deployed to communicate with the imaging diagnostic database and to perform a diagnosis task for schizophrenia classification along with brain morphometry identification. Apart from the back-end, the front-end interface is composed of JavaScript (js) and WebGL with plug-in applications “Papaya” in Mango image processing software (Lancaster, Martinez; www.ric.uthscsa.edu/mango) for visualization of brain structural abnormality.

## Algorithm Validation and Statistical Analysis

Six quantities are reported in this study for validating the EDNN algorithm: specificity, sensitivity, accuracy, precision (PPV), negative predictive value (NPV) and Number Needed to Predict (NNP) (10). To assist with parameter tuning, we validated these quantities and the receiver operating characteristic (ROC) curves on two independent cohorts using the EDNN framework, which gives an estimate of diagnostic utility and how well the model will perform in a new dataset.

## Results

### Classifier Performance

**Figure 3:**
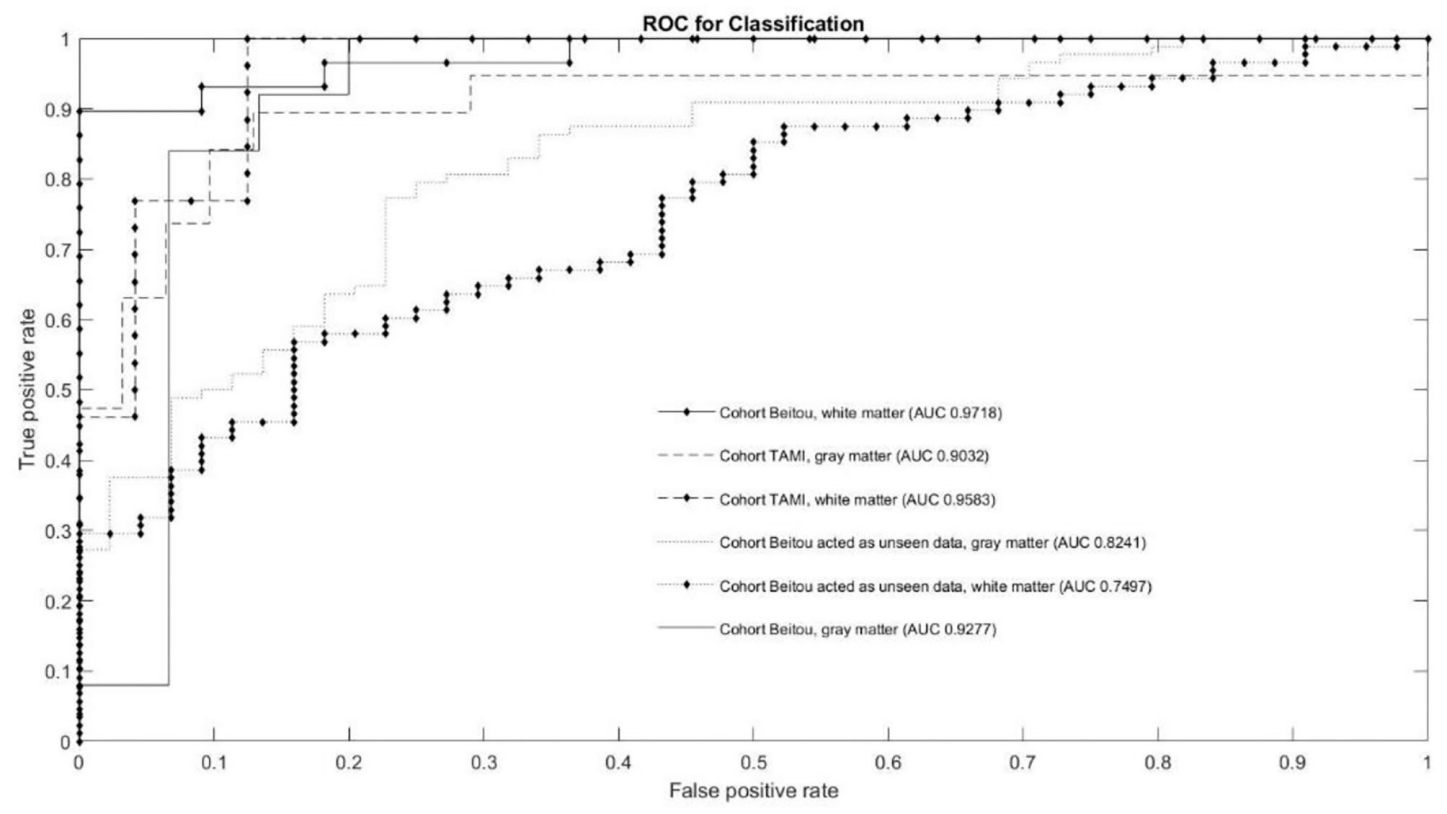
Quantitative performance of our model in ROC curves and AUC scores.

The classification results of the models are given in Table 3. Through application of the EDNN on both TAMI (400 subjects) and Beitou (132 subjects) cohorts, the training accuracy reached 100.00 % in the classification of schizophrenia and healthy controls. On the other hand, the EDNN classifier discriminated schizophrenia cases and healthy controls with 84.00% (95% confidence interval CI: 73.97–84.36) accuracy and 90.22% (95% CI: 85.40–91.40) accuracy for GM and WM, respectively in the TAMI cohort. Using the Beitou cohort, it yielded classification accuracy of 77.27% (95% CI: 75.23–81.32) for GM and accuracy of 70.45% (95% CI: 68.58–74.29) for WM. After performing transfer learning by retraining the model, it achieved 85.50% (95% CI: 85.23–88.07) for GM and accuracy of 88.20% (95% CI: 86.52–88.80) for WM. The ROC curves in Figure 3 showed the area under the curve (AUC) scores indicating the competitive performance using our models.

**Table 3:** The performance of the EDNN model in two independent cohorts with GM and WM imaging data.

**Cohort—tissue**	**Accuracy (95% CI)**	**Specificity (95% CI)**	**Sensitivity (95% CI)**	**PPV**	**NPV**	**NNP**
TAMI-GM	84.00 (73.97–84.36)	80.62 (68.31–90.71)	89.47 (66.49–89.97)	73.91	80.65	1.8329
TAMI-WM	90.22 (85.40–91.40)	91.23 (80.70–93.54)	89.21 (86.7–93.98)	86.67	83.33	1.4286
Beitou-GM	85.50 (85.23–88.07)	72.12 (65.45–72.21)	95.25 (94.46–97.04)	89.29	80.00	1.4433
Beitou-WM	88.20 (86.52–88.80)	72.30 (65.90–72.51)	96.55 (96.37–97.94)	90.32	72.73	1.5860
Beitou-GM-unseen	77.27 (75.23–81.32)	65.91 (62.03–72.32)	82.95 (72.82–87.62)	82.95	65.91	2.0465
Beitou-WM-unseen	70.45 (68.58-74.29)	52.27 (51.28–54.29)	79.55 (71.82–80.28)	76.92	52.27	3.4251

### Framework of Web Diagnostic System

As the framework is illustrated in previous section, the EDNN produced a classifier for the AI-based diagnostic system from our cohorts, and the web-platform provided a fast and easy access to the machine-learning technique we developed. Taken together, we presented an online AI diagnostic system (www.brain-diagnosis.com) which is a fully open cloud service to both clinical and academic end users, aiming to help classify and diagnose schizophrenia patients via the internet.

**Figure 4:**
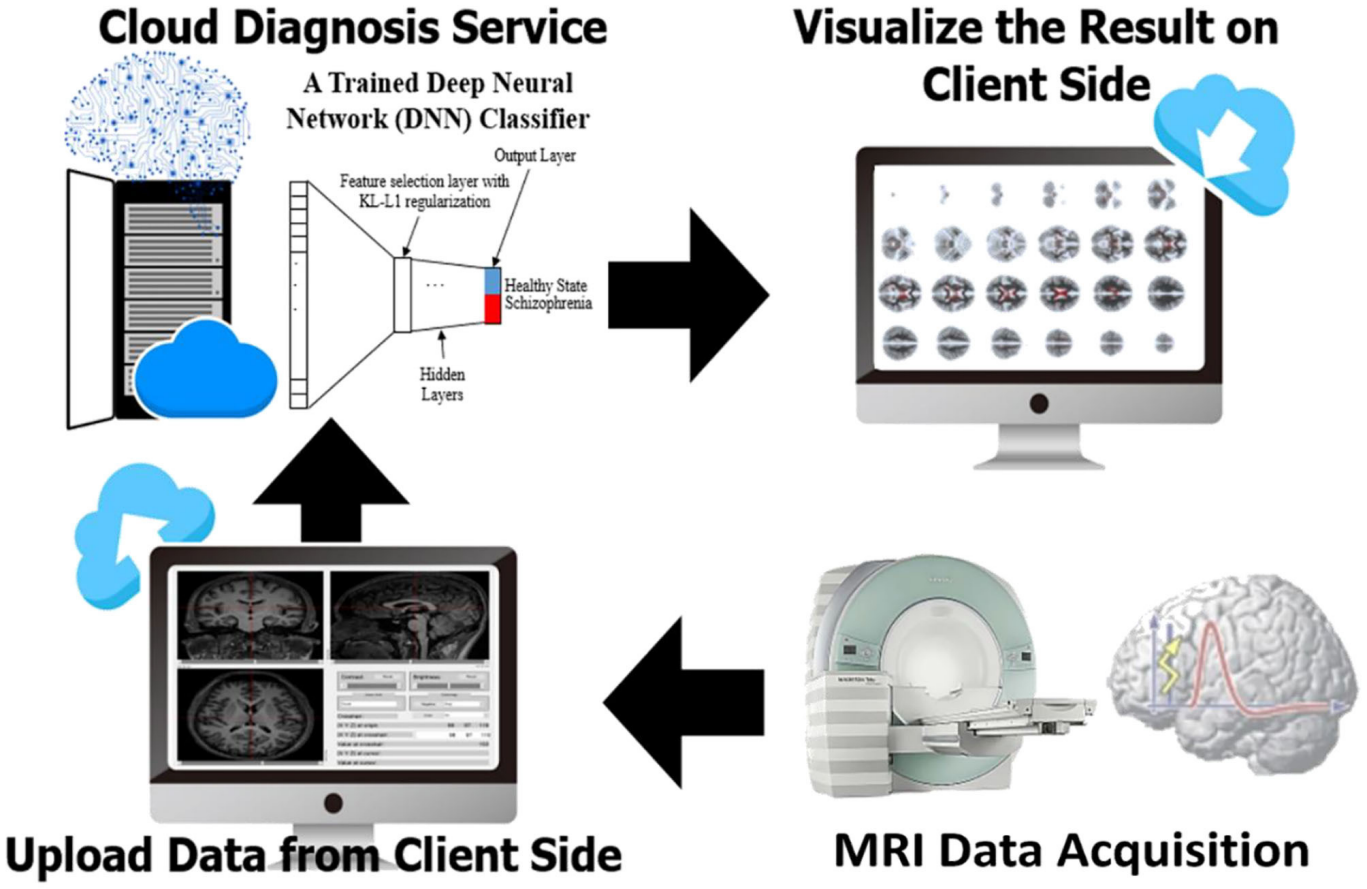
Schematic depiction of our prediction and evaluation workflow.

Figure 4 illustrates a schizophrenia classification and diagnostic result using our AI-based system. When the end user uploads a structural MRI image to our web platform, the backend will perform the following tasks. Brain tissues necessary to perform voxel-based morphometry are first extracted from the DICOM image taken by an MRI scanner. The task of classification and resulting diagnosis are presented on the diagnostic system. The diagnostic system is capable of converting all the brain image data and diagnosis result for graphical preparations and visualizations through the user's local computing resources for the graphical abilities. The end-user can simply upload the NIFTI files of GM and WM images, and then the classification of schizophrenia along with pragmatic diagnosis will be delivered in a browser instantaneously.

Furthermore, our web platform is capable of visualizing the deficits of voxel morphometry identified by the EDNN model. A voxelwise brain volume map of the GM densities was transformed to the z-score with respect to the MRI data in our cohorts.

$$\begin{array}{l} \text{ zscore}\_\text{HC}=\;\frac{\text{value of voxel}-\text{mean value of voxels from HC}}{\text{std value of voxels from HC}}\\ \text{zscore}\_\text{SCZ}=\;\frac{\text{value of voxel}-\text{mean value of voxels from SCZ}}{\text{std value of voxels from SCZ}}\\ \text{ z-score}=\;\frac{\text{zscore}\_\text{SCZ}}{\text{zscore}\_\text{SCZ}+\text{zscore}\_\text{HC}} \end{array}$$

**Figure 5:**
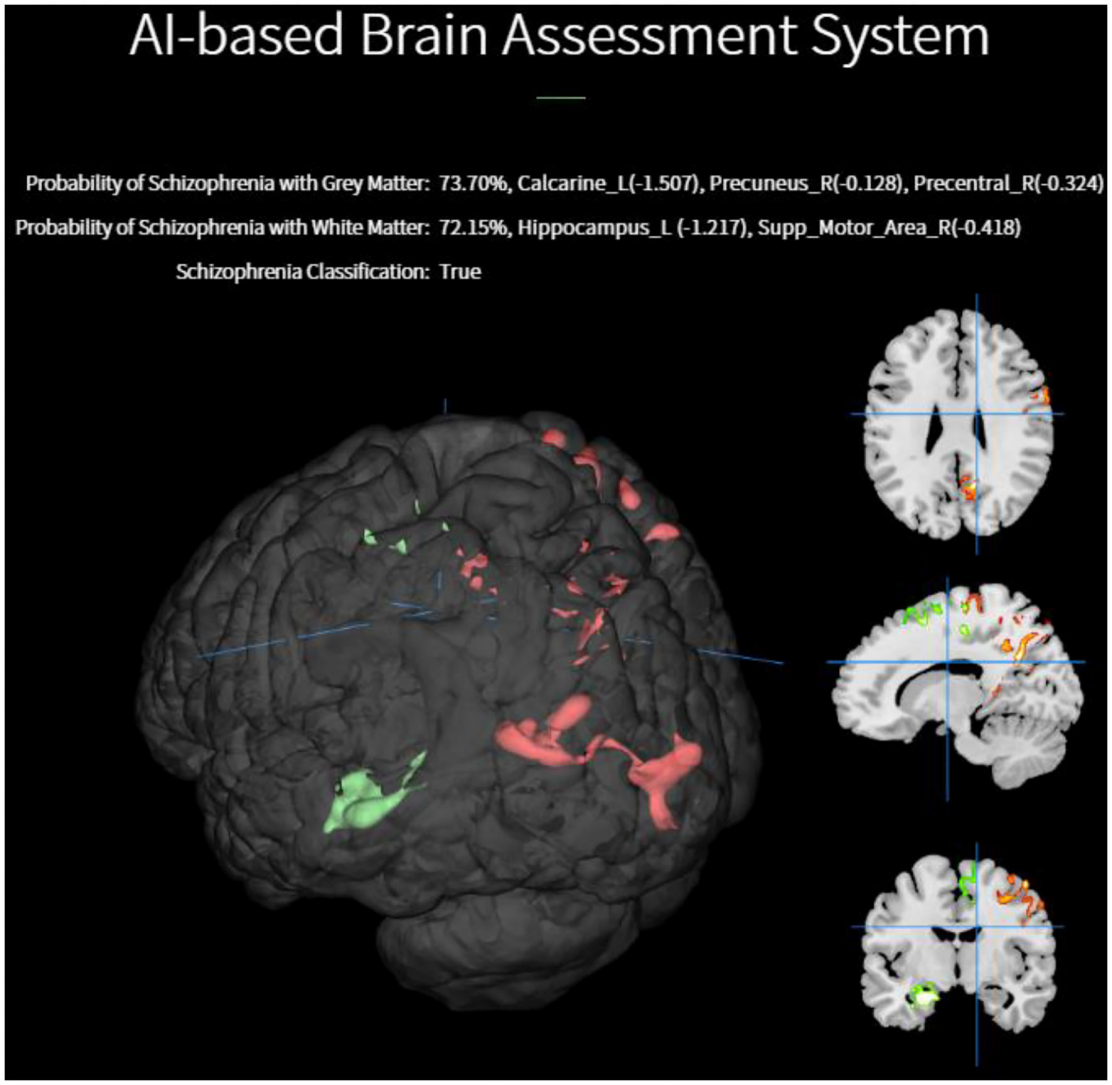
Individualized visualization and z-score analysis delivered by our diagnostic platform for which the volumes were significantly reduced in a single schizophrenia patient compared with healthy subjects.

In Figure 5, a 3D saliency map of one demonstrative case with the highlighted brain regions shows significant GM reduction in the insula, anterior, and anterior cortex. In doing so, the platform based on the EDNN model gives us a new straightforward approach on the automated individual-level assessment of schizophrenia.

## Discussion

### Summary of Findings

Adopting EDNN to investigate schizophrenia shows a practical advantage without requiring manual feature selection as other conventional machine learning approaches do. With the aim to preserving the 3D structure in the classification pipeline and elucidating the neural correlates of mental disorders, it is critical both to identify schizophrenia and to find relevant brain-based imaging markers simultaneously. In line with the thought, we applied an EDNN model to visualize the brain structural deficits and to distinguish patients with schizophrenia from healthy controls in two large independent samples using structural MRI data.

EDNN is a particular type of the deep neural network showing its data-driven interpretation along with promising recognition accuracy. The strength of our approach was its novel use of an EDNN model to distinguish patients with schizophrenia from healthy controls without anatomical priors. The model's discriminative pattern showed decreased GM density in the insula and frontal and superior temporal lobes as well as reduced intensity of WM in the cingulum/hippocampus, splenium of corpus callosum, and posterior corona radiate tracts. These deep-learning-based findings provide important brain-based imaging markers for subsequent neuroimaging analyses and the development of our web platform.

### Evaluation of Our Results With Others

The 89.63% average classification accuracies of our model obtained in two large independent cohorts were relatively compelling, compared to relevant studies in literature (8–10, 19). Besides, the AUC score indicates that EDNN performed well for classifying patients from controls (19). The current finding of NNP suggests that the EDNN classifier is capable of providing reproducible results across different large cohorts. Even though some studies (7, 12, 26, 27) asserted the decrease in accuracy being attributed to greater variability in brain endophenotypes with sample size increase, the EDNN framework in this study appears to be promising in discrimination of schizophrenia by integrating rich information in VBM.

### Research Domain Criteria

Visualization of the salient brain region provides important clinical information by using our AI-based diagnostic platform. The rationale as “explainable” deep neural network might aid in the development of neuroimaging biomarkers for psychiatric disorders. As suggested by the Research Domain Criteria (RDoC) approach, our approach is directly relevant to the search for biomarkers because our framework yields more robust feature representation and reliable performance on classification. Therefore, we expect this web-based RDoC approach may improve the reliability of psychiatric diagnosis as well as the objectiveness of clinical evaluation.

An increasing amount of neuroimaging data has been established in recent years to seek for machine learning-based classification of neuropsychiatric diseases, such as Autism Brain Imaging Data Exchange (28), Alzheimer's disease neuroimaging initiative (29), or Bipolar & Schizophrenia Network on Intermediate Phenotypes (30). These databases have been released to the public or have plans to be public in the near future, facilitating the process of developing AI-based neuroimaging analysis. Future validation of our EDNN method with related and larger databases that contains heterogeneous cohorts could help evaluate the adaptability and improve the performance of our web-based diagnostic platform. Furthermore, our EDNN approach has identified certain brain regions related to schizophrenia, which could help generate hypothesis-driven research of pathophysiology of schizophrenia. These brain-based imaging markers may augment the doctor to improve the decision-making and assessment process in the workflow of the clinical practice. Importantly, this EDNN algorithm is accessible publicly through our web-based diagnostic platform.

### Limitations

There are several limitations to our study. First, it should be noted that the imbalanced dataset and different cohorts might have affected the performance when using the model. While our model achieved approximately 90% within a single cohort, it became <75% when we applied the trained model with a completely unseen dataset (i.e., Beitou cohort). Furthermore, the uneven number of classes, i.e., 88 schizophrenia and 44 healthy controls, might have also caused the drop of predictive performance. To overcome the issue, data sampling and ensemble system may be employed (31). Note that with the two approaches, altering the distribution of the training data may cause biasing.

Second, psychosis is still a heterogeneous entity. Although our model can get promising accuracy in the binary classification, it is difficult to directly generalize the mutual information analysis to a multi-class, multi-label classification task across other psychotic disorders such as schizoaffective, depression, and bipolar disorders.

Therefore, further investigation may be focused on generalization to other neuropsychiatric disorders (e.g., Alzheimer's disease, Parkinson's disease). In addition, more examination is needed on the feasibility of identifying biological subtypes of psychotic disorder. By using our web-based platform, we aim to come up with persuasive phenotypes based on the brain anatomic abnormalities.

## Conclusion

This paper presented a new platform for a publicly available brain diagnostic system over browsers called “The AI-based online brain diagnosis system” which shows the potential diagnostic ability for schizophrenia using a T1-weighted image. This tool is to provide an objective quantification of brain pathology rather than to replace the conventional diagnostic interview in the clinical practice.

Schizophrenia is a heterogeneous disorder with respect to the involvement of many co-occurring symptoms. Traditionally, the diagnosis of schizophrenia is mainly based on symptom profile with descriptors of the course of illness. Yet, there have been cases that missed to be identified with such approach. More generally, it has long been a debate on the underlying principles of diagnosing schizophrenia. The platform proposed in our study is a proof of concept by demonstrating the use of deep-learning models to differentiating schizophrenia from healthy controls based solely on imaging markers. We anticipate that this platform can be generalized to the differential diagnosis of multiple psychiatric disorders such as schizophrenia, bipolar disorder, or unipolar depression. The main motivations for developing this platform are to facilitate objective diagnosis by the user-friendly website interface and to promote the visibility and availability of newly developed state-of-the-art algorithms for the diagnosis of schizophrenia. An overview of the main user interface is currently available on “brain-diagnosis.com.” We envisage that this platform can bring improvements in the effectiveness and impact of the deep learning applications in neuroimaging.

## Data Availability Statement

The datasets generated for this study are available on request to the corresponding author.

## Ethics Statement

The studies involving human participants were reviewed and approved by the Institutional Review Board in Taipei Veterans General Hospital, National Yang-Ming University and Tri-Service Military General Hospital, Beitou Branch. The patients/participants provided their written informed consent to participate in this study.

## Author Contributions

Y-WC: manuscript writing, programming, and data analysis. S-JT: manuscript discussion and statistical analysis. Y-FW: study design, participants selection, and collection. AY: study design, data collection, development of EDNN model, and supervision of the project.

## Conflict of Interest

The authors declare that the research was conducted in the absence of any commercial or financial relationships that could be construed as a potential conflict of interest.
